# Acute Vestibular Neuritis After Pfizer Booster in a Healthcare Worker

**DOI:** 10.7759/cureus.24668

**Published:** 2022-05-02

**Authors:** Raya Aliakbar, Antonio K Liu, Juan Barrio

**Affiliations:** 1 Neurology, Adventist Health White Memorial, Los Angeles, USA; 2 Internal Medicine, Adventist Health White Memorial, Los Angeles, USA

**Keywords:** reversible, health-care worker, covid 19, pfizer booster, vestibular neuritis

## Abstract

Numerous reports of healthy individuals falling ill after COVID-19 vaccination or booster have surfaced. Isolated vestibular dysfunction is uncommon. Such occurrence within 24 hours of booster shot in a relatively healthy highly functional colleague suggests beyond a simple temporal relationship.

## Introduction

COVID-19 has been declared a pandemic by the WHO since March of 2020. In the United States, there have been 78.8 million cases and 947,882 deaths [1]. Besides isolation precautions and masks, vaccinations have emerged as the most prolific and effective way of preventing serious complications of COVID-19.

Among the first to get vaccinated were healthcare workers and first responders. The first available vaccine schedule in the United States consists of two doses of mRNA-based vaccination, spaced three weeks apart for Pfizer and four weeks apart for Moderna. With the emergence of variants and waning immunity, concerns were raised especially for healthcare workers, as they are disproportionately exposed to COVID-19. Thus, booster vaccinations became a novel solution to the emerging problem. While side effects for the initial two doses of vaccinations are well studied, there is scant data regarding the side effects of booster vaccines in patients. A case study in July 2021 reported a 56-year-old doctor who had pain, pruritus, and erythema at her injection site, called “COVID arm” one week after her booster shot of Moderna [2]. However, data regarding the adverse effects of the three-dose vaccine series is not well studied. In this case study, we present a 40-year-old male with symptoms of ataxia, and vestibular neuritis 24 hours following his booster shot of the Pfizer vaccine.

## Case presentation

**Figure 1:**
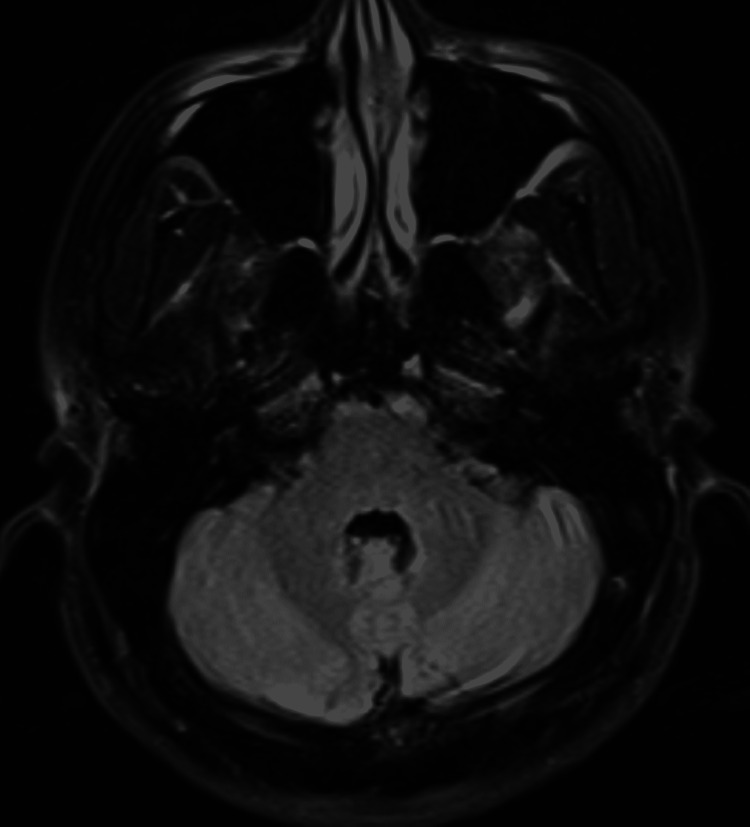
FLAIR of head MRI showing normal posterior fossa.

The patient is a 40-year-old male internist with a past medical history of eczema as a child and newly diagnosed psoriatic vs. reactive arthritis in early 2020. The patient presented to ED complaining of nausea, lightheadedness, and poor coordination one day following the administration of the Pfizer COVID booster shot. The patient received his second dose of the Pfizer vaccine in January 2021 with no symptoms other than fatigue. He denied a prior confirmed diagnosis of COVID-19. On arrival, the patient’s mentation was intact. The cranial nerve exam was unremarkable with no extraocular movement deficit and no nystagmus. He had intact strength and sensation. There was no dysmetria or dysdiadochokinesia on finger-to-nose examination. However, when he stood up, he had truncal ataxia, a broad base slow gait. he could not perform a tandem walk. His vital signs were normal. He was afebrile. Hematology results showed WBC of 5.2K/µL, HgB of 13.8 g/dL, HCT of 41.5%, and PLT of 220K/µL. Chemistry results showed sodium of 136 mmol/L, potassium of 4.1 mEq/L, BUN of 12 mg/dL, creatinine of 0.8 mg/dL and GFR of 106 mL/min/1.73m^2^. MRI brain was negative and nonrevealing (Figure 1). The patient received meclizine and Zofran in the ED, and the patient endorsed that meclizine did help with vertigo. He was discharged home for recuperation. Daily follow-up phone calls were performed by the neurologist. For the following five days after discharge from Emergency Department, the patient could not drive, eat, watch TV or walk without experiencing vertigo or truncal ataxia. While his symptoms continued to improve over time, it took the patient five to six days to return to his baseline.

## Discussion

In this case study, we present a case of vestibular neuritis, 24 hours after the administration of the Pfizer booster vaccine with the resolution of major symptoms by seven days. Common side-effects that have been reported with the Pfizer vaccine are non-severe allergic reactions such as rash, itching, hives, swelling of the face, myocarditis, injection site pain, fatigue, headache, chills, fever, nausea, vomiting, lymphadenopathy, and diarrhea [3]. More recently, data released from the FDA for licensure of the Pfizer Booster vaccine showed that during phase 2/3 trials, 1.6% or five participants had a sort of nervous system disorder, and 5.2% or 16 participants had lymphadenopathy [4]. On October 21, 2021, Pfizer announced data from their phase 3 randomized control trial evaluating the efficacy and safety of a 30-µg booster dose of the Pfizer vaccine in 10,000 patients 16 years and older. During the study period (seven days after booster to 2.5 months) there were five cases of COVID-19 in the booster group compared to 109 in the non-boosted group. Therefore, the relative vaccine efficacy was 95.6% compared to those who did not receive a booster [5].

Thus, with emerging data indicating the efficacy of booster vaccines, side effects of such should be thoroughly investigated. In this case study of a patient with vestibular neuritis and possible association to mRNA-based booster vaccine (Pfizer), it can be theorized that the increased immune reaction to receiving the booster incited an auto-immune response, affecting the vestibular nerve and thus creating symptomatology of vertigo and altered proprioception. As the patient denies any tinnitus or hearing loss, vestibular neuritis is more likely than labyrinthitis.

To note, this patient has a past medical history of auto-immune rheumatological issues newly arising in 2020. He reports that at the beginning of 2020 he had weight loss and migratory joint pain, from shoulder to elbow, sternum, and then hip unilaterally as well as dactylitis. When seen by a rheumatologist, he was found to have very high ESR and CRP levels with negative ANA titer and was diagnosed with reactive arthritis vs. psoriatic arthritis. After migratory arthritis, the patient also developed patchy inflammation, specifically on the scalp, which continued even after the sequelae of the booster vaccine. It could therefore be theorized that patients with previously diagnosed auto-immune conditions, such as reactive arthritis in this patient, have a greater predisposition to enhanced inflammatory responses from mRNA-based booster vaccinations.

## Conclusions

While we suspect such association of vestibular arthritis following mRNA-based booster (Pfizer) is beyond temporal as reported in this case study, it is only through systemic reporting and analysis that further conclusion and pathophysiology can be determined. As far as we can tell, the reversibility and mild nature of such an event did not deter our colleague's determination to receive future booster vaccines.
